# Revealing the significant shortcomings in the learning environment at the three largest medical schools in Syria: what’s next?

**DOI:** 10.1186/s12909-022-03978-4

**Published:** 2023-01-03

**Authors:** Ghaith Alfakhry, Ahmad Naeem, M. Bader AboHajar, Aisha Alfakhry, Abdul Fattah Mohandes, Iyad Ali, Ebrahim Makhoul, Nadeem Ahmed, M. Mhdy Abla, Khaled Alhomsi, Issam Jamous

**Affiliations:** 1 Education Quality and Scientific Research Office, Al-Sham Private University, Damascus, Syria; 2 Faculty of Dentistry, Damascus University, Damascus, Syria; 3 Program of Medical Education, Syrian Virtual University, Damascus, Syria; 4 Faculty of Medicine, Damascus University, Damascus, Syria; 5 Faculty of Medicine, University of Aleppo, Aleppo, Syria; 6 Faculty of Medicine, Tishreen University, Latakia, Syria; 7 Cancer Research Center, Tishreen University, Latakia, Syria; 8 Department of Internal Medicine, Tishreen University Hospital, Latakia, Syria; 9 Department of Biomedical Sciences, Al-Sham Private University, Damascus, Syria; 10 Department of Prosthodontics, Faculty of Dentistry, Damascus University, Damascus, Syria

**Keywords:** Learning environment, Medical education, DREEM, Syria

## Abstract

**Background:**

Medical education in Syria still adopts a traditional, teacher-centered curriculum to this day. These elements imply the existence of issues in the learning environment (LE). This study aims to provide the first evaluation of the LE at the largest medical schools in Syria using the DREEM inventory.

**Methods:**

The three largest medical schools in Syria are the ones at Damascus University (DU), University of Aleppo (AU), Tishreen University (TU). The Arabic version of the DREEM questionnaire was used. Students across all years of study except year 1 were approached. Both paper-based and electronic surveys were conducted.

**Results:**

A total of 1774 questionnaire forms were completed (DU:941, AU:533, TU: 300). The overall DREEM score at DU, AU, and TU were 100.8 ± 28.7, 101.3 ± 31.7, and 97.8 ± 35.7 respectively with no significant difference (*P* = 0.254) between the three universities. DREEM subscales concerning *Learning, Atmosphere, Academic Self-perception and Social Self-perception* had a low score across all universities. Clinical-stage students reported significantly lower perception (*P*** ≤ **0.001) of the LE in comparison to their pre-clinical counterparts across all subscales.

**Conclusions:**

The findings of this study highlight the significant shortcomings of the medical LE in Syria. If not addressed properly, the academic, clinical, and professional competence of the healthcare workforce will continue to deteriorate. Moreover, the negative LE might be a predisposing factor for medical students’ exodus. The Syrian medical education system requires leaders who are willing to defy the status quo to achieve a true educational transformation.

**Supplementary Information:**

The online version contains supplementary material available at 10.1186/s12909-022-03978-4.

## Background

The practical ramifications of a suboptimal learning environment (LE) are far-reaching in medical education. Not only it could hamper students’ academic development [1], but it could also negatively affect their professional attitude and hence their values and identity as future physicians [2, 3]. In line with the wisdom of “what gets measured, gets managed”, researchers developed several methods and tools to evaluate and identify areas of improvement in the medical learning environment. The Dundee Ready Educational Environment Measure (DREEM) inventory is considered the most used tool to evaluate the LE globally; it has established validity and reliability and has been translated to different languages including Arabic [4–6]. Although the DREEM inventory provides a quantitative measure of the LE, a previous study supported the notion that DREEM studies can provide a meaningful qualitative assessment equal to that of extensive and laborious qualitative interviews [7].

Medical education in Syria has been using the same traditional teacher-centered curriculum for over decades now [8]. The curriculum is overloaded and fragmented with no vertical nor horizontal integration; instead of problem-based learning, medical schools still depend on an information-gathering approach; the main method of instruction is large-group teaching through lectures [9]. Although small-group teaching is available during clinical rotations, teaching hospitals do not follow a certain teaching structure during clinical rotations. In many cases, students are left unattended to wander around the hospital with no clear objective. Many students opt for leaving after taking attendance making the clinical rotations empty of educational value. As for assessment methods, multiple choice questions and unstructured oral exaiminations are the primary methods of assessing students’ acquisition of knowledge and clinical skills. To this day, there hasn’t been an adoption of any of the widely used clinical assessment methods such as OSCE (Objective Structured Clinical Examination), DOPS (Direct Observation of Procedural Skills), and mini-CEX (Mini Clinical Evaluation Exercise), etc. Further, formative assessment is not part of the curriculum. All these elements are indicative of an unfavourable learning environment. Previous DREEM studies demonstrated that medical schools with a traditional curriculum had low DREEM scores in comparison to medical schools that use modern innovative student-centered curriculum [10, 11]. In the middle east, two traditional medical schools in King Abudl Aziz University, Saudi Arabia and Sana’s University, Yemen scored low on the DREEM scale (102/200 and 100/200 respectively) in comparison to Dundee Medical School in the UK (139/200) that adopts a more modern outcome-based curriculum [10]. Less psychological distress and better social support have also been associated with a better perception of the LE [12].

In Syria, recent studies showed significant psychological disturbances among medical students [9, 13]. The calamities brought by the ongoing Syrian war have affected the lives of the Syrian population on all levels including social, economic as well as educational [14]. Classes in universities in conflict zones were suspended, and many students were transferred to other universities in conflict-free areas such as Damascus University [9]. This placed a huge burden on the hosting universities which teaching capacity was already failing short due the emigration of the teaching staff; the teaching facilities have also not been designed to accommodate this huge number of students [8, 15, 16]. Moreover, medical schools have been greatly affected by the economic crisis: teaching staff is in short supply and are underpaid, development and renovation projects are ceased and teaching hospitals are in need of rehabilitation that is yet to occur [8, 9, 17].

Even though the Syrian war-torn context along with the traditional curriculum at medical schools have major negative implications on the LE, no previous attempts have been made to assess the LE at medical schools. Hence, it is this study’s aim to conduct the first large-scale evaluation of the learning environment at medical schools in Syria, providing an insight into its areas of improvement and making suggestions to approach major issues effectively.

## Methods

Ethical approval for the study has been granted by the Syrian Virtual University (SVU) no. 479/0 on April 19th, 2022.

The curriculum at medical schools is standardized in Syria according to the recommendations and guidelines of the Ministry of Higher Education and Scientific Research. It is comprised of 6 years: 1 preparatory year, 2 pre-clinical years, and 3 clinical years. The preparatory year has been introduced in 2015 for those who want to study medicine, dentistry, or pharmacy. After completing the sixth year, medical students need to pass the Unified National Medical Examination (UNME) to obtain their medical degree. The UNME is a multiple-choice question exam that has been in effect since 2012 [9]. Upon graduation, students start their residency in a certain specialization based on their GPA and UNME grade.

There are seven public medical schools in Syria. The current study included the three largest and oldest medical schools in Syria which are Damascus University (DU), University of Aleppo (AU), and Tishreen University (TU), located in Damascus (the capital, southwestern region), Aleppo (northwestern region) and Latakia (western region) respectively. These three medical schools host the majority of medical students in Syria. Regarding the year of study, the defined population at these three medical schools included all students across all years of study except for the 1^st^ preparatory year which was excluded as these 1^st^-year students may opt to study dentistry or pharmacy instead of medicine.

The Arabic version of the validated 50-statement DREEM instrument was used to examine students’ perception of the LE [18] (Supplementary file 2). Each statement is scored on a 5-point scale: strongly disagree (0), disagree (1), unsure/inapplicable (2), agree (3), and strongly agree (4). The DREEM inventory has five subscales: students’ perception of learning, students’ perception of teachers, students’ academic self-perception, students’ perception of the atmosphere, and students’ perception of the social environment. According to previous recommendations [19], a grading rubric was used to interpret the DREEM scale and its five subscales (Table 1). Both paper-based and electronic surveys were administered Online surveys were used to comply with certain universities policies. Whereas, paper-based surveys were utilized when university policy facilitated it. In the paper-based survey, convenience and snowball sampling methods were used, whereas online surveys depended on river sampling in addition to convenience and snowball sampling. The data collection process was carried out in the second term between April 21^st^, 2022 and July 31^st^, 2022. All incomplete DREEM forms were omitted from the final analysis.

**Table 1:** A guide for interpretating the DREEM scale and its subscales

	**Score**	**Interpretation**
**Total DREEM**	0–50	Very poor
	51–100	Plenty of problems
	101–150	More positive than negative
	151–200	Excellent
**Subscales**		
**Learning**	0–12	Very poor
	13–24	Teaching is viewed negatively
	25–36	A more positive approach
	37–48	Teaching highly thought of
**Teachers**	0–11	Abysmal
	12–22	In need of some retraining
	23–33	Moving in the right direction
	34–44	Model teachers
**Academic self-perception**	0–8	Feeling of total failure
	9–16	Many negative aspects
	17–24	Feeling more on the positive side
	25–32	Confident
**Atmosphere**	0–12	A terrible environment
	13–24	There are many issues that need to be changed
	25–36	A more positive atmosphere
	37–48	A good feeling overall
**Social self-perception**	0–7	Miserable
	8–14	Not a nice place
	15–21	Not very bad
	22–28	Very good socially

Negatively-phrased items (statements) were coded in reverse so that the higher the score, the more positive the perception is; those items were: no. 4, 8, 9, 17, 25, 35, 39, 48, and 50. Cronbach’s Alpha was used to measure the internal consistency of the DREEM instrument. Man-Whitney U test was administered to compare every two independent groups as the Shapiro Wilk test was significant. One-way ANOVA was used to compare the three universities; Games-Howell post-hoc was used as the Levene normality test *p*-value was < 0.05. Eta squared was used as a measure of effect size. A reduced *p*-value of < 0.01 was adopted to countermeasure the risk of mass-significance when comparing three groups [20]. In all other cases, a *p* < 0.05 was considered significant. Google Forms was used to administer the electronic survey; Microsoft Excel 2019 was used to process the data; IBM SPSS Statistics for Windows, version 26 (IBM Corp., Armonk, N.Y., USA) was used to perform statistical analysis. Data collected and/or generated during the current study are available on figshare [21].

## Results

A total of 1774 students completed the DREEM form. The number of respondents recruited via online surveys at DU, AU, and TU was 256, 533, 265 respectively; the rest were recruited via paper-based surveys. Table 2. shows the demographic information and size of the sample and its respective population at the three selected universities. The sample-population ratio was 15.5% at DU, 10.9% at AU, and 6.4% at TU. Cronbach’s Alpha value for the 50 statements was 0.928. The Cronbach’s Alpha values for each subscale is provided in Table 6, Supplementary file 1.

**Table 2:** Demographic information of participants at each university

	**Damascus University**		**University of Aleppo**		**Tishreen University**	
	Populatio*n* size	Sample size	Populatio*n* size	Sample size	Populatio*n* size	Sample size
**Year of study**						
2^nd^ year	1000	169	878	129	920	25
3^rd^ year	1165	157	894	190	932	96
4^th^ year	1116	173	901	60	935	86
5^th^ year	1224	245	1141	107	915	67
6^th^ year	1536	197	1043	47	950	26
Total	6041	941	4857	533	4652	300
**Female %**	-	51.5% (*n* = 485)	-	47.3% (*n* = 252)	-	48.3% (*n* = 145)
**Age mean (SD)**	-	22.0 (2.04)	-	21.4 (1.6)	-	21.5 (1.3)

*SD* Standard deviation

The mean scores of the overall DREEM scale and its subscales at each university are provided in Table 3. Concerning the overall DREEM score, no significant difference between the three universities was detected. Except for the *Teachers* domain, the mean scores of all subscales at the three universities indicated a negative perception. There was a significant difference between DU and the other two universities in the *Teachers* subscale with a small effect size. The lowest scoring domain was the *Social Self-perception* (*n* = 1774, 43.5%) followed closely by Learning (*n* = 1774, 45.0%).

**Table 3:** Descriptive statistics of the DREEM scale and subscales at each university with One-way ANOVA analysis

	**DU (*n* = 941)**	**AU (*n* = 533)**	**TU (*n* = 300)**	**Total (*n* = 1774)**	**One-way ANOVA**		
	Mean ± SD	Mean ± SD	Mean ± SD	Mean ± SD	*p*-value	Eta squared	Post-hoc
**Learning (max. 48)**	21.5 ± 7.9	22.3 ± 8.3	20.6 ± 9.2	21.6 ± 8.3	0.012	0.005	AU/TU
**Teachers (max. 44)**	27.0 ± 7.8	25.2 ± 8.5	25.4 ± 9.2	26.2 ± 8.3	< 0.001	0.011	DU/AU, DU/TU
**Academic self-perception (max. 32)**	16.6 ± 6.0	16.6 ± 6.2	16.2 ± 6.8	16.5 ± 6.2	0.643	< 0.001	-
**Atmosphere (max. 48)**	23.4 ± 8.5	24.6 ± 9.2	23.1 ± 10.6	23.7 ± 9.1	0.038	0.004	-
**Social self-perception** **(max. 28)**	12.0 ± 4.6	12.4 ± 5.0	12.2 ± 5.2	12.2 ± 4.8	0.392	0.001	-
**DREEM** **(max.200)**	100.8 ± 28.7	101.3 ± 31.7	97.8 ± 35.7	100.4 ± 30.9	0.254	0.002	-

Eta squared is an indicator of effect size. Underlined cells indicate a value of negative interpretation

Table 4 illustrates the DREEM scale and subscales scores according to the year of study, stage (pre-clinical, clinical), and sex. In terms of year of study, there was a significant difference (*P* < 0.001) in the DREEM score according to One-way ANOVA. Post-hoc Tukey showed that Year 4, 5, 6 students scored significantly less on the DREEM scale (*P* ≤ 0.001) than their Year 2 and 3 counterparts. Year 2 students perceived the *Learning, Academic Self-perception, and Social Self-perception* negatively, whereas their perception of *Teachers* and *Atmosphere* was positive. The overall DREEM score for second year students was 106.3 ± 30.3. In comparison, year 6 students perceived all subscales negatively and the overall DREEM score was 96.5 ± 30.4. Similarly, there was a significant difference between pre-clinical and clinical students in *Academic Self-perception* (*P* = 0.002) and across all other subscales (*P* < 0.001) and in the overall DREEM score (*P* < 0.001). In terms of sex, male students scored significantly lower on the *Teachin*g (*P*** < **0.001), *Atmosphere* (*P* = 0.024), and the overall DREEM score (*P* = 0.03).

**Table 4:** DREEM scale and subscale findings according to the year of study, stage and sex

	**Year of study**					**Stage**		**Sex**	
	Year 2 (*n* = 323)	Year 3 (*n* = 443)	Year 4 (*n* = 319)	Year 5 (*n* = 419)	Year 6 (*n* = 270)	Pre-clinical (*n* = 766)	Clinical (*n* = 1008)	Male (*n* = 892)	Female (*n* = 882)
**Learning (max. 48)**	23.6 ± 8.1	23.2 ± 8.0	19.9 ± 8.6	20.4 ± 7.9	20.4 ± 8.3	23.4 ± 8.0	20.2 ± 8.2	21.2 ± 8.3	22.0 ± 8.3
**Teachers (max. 44)**	27.1 ± 8.3	28.0 ± 8.2	25.6 ± 8.1	25.0 ± 8.3	24.5 ± 8.1	27.6 ± 8.2	25.1 ± 8.2	25.3 ± 8.5	27.1 ± 8.0
**Academic self-perception (max. 32)**	16.8 ± 6.2	17.3 ± 6.0	15.9 ± 6.6	16.1 ± 6.0	16.5 ± 6.4	17.1 ± 6.1	16.1 ± 6.3	16.7 ± 6.3	16.4 ± 6.1
**Atmosphere (max. 48)**	25.8 ± 8.5	25.8 ± 8.8	21.7 ± 9.3	22.0 ± 8.9	22.8 ± 9.2	25.8 ± 8.7	22.1 ± 9.1	23.2 ± 9.1	24.2 ± 9.1
**Social self-perception** **(max. 28)**	12.8 ± 4.9	12.6 ± 4.9	11.5 ± 4.9	11.8 ± 4.8	12.1 ± 4.3	12.7 ± 4.9	11.8 ± 4.7	12.0 ± 4.7	12.4 ± 4.9
**DREEM** **(max.200)**	106.3 ± 30.3	107.1 ± 30.0	94.8 ± 31.9	95.5 ± 30.0	96.5 ± 30.4	106.8 ± 30.1	95.6 ± 30.7	98.6 ± 31.3	102.2 ± 30.5

Underlined cells indicate a value of negative interpretation

The DREEM items (statements) which scored less than 2.00 on the disagreement/agreement scale are shown in Table 5. Items that score below 2 indicate a more negative than a positive response. At the individual item level, the number of statements that had a mean score below 2.00 was 28 (56%), 28 (56%), and 25 (50%) at DU, AU, and TU respectively. Only two items at each university scored over 3.00. The universities had similar mean scores in the majority of statements. Descriptive statistics of each DREEM statement are available in Table 8, Supplementary file 1.

**Table 5:** Mean score of each DREEM statement that had a negative interpretation (< 2.00) at each university

**Items according to their subscales**	**Damascus University** (*n* = 941)	**University of Aleppo** (*n* = 533)	**Tishreen University** (*n* = 300)
	Mean ± SD	Mean ± SD	Mean ± SD
**Students’ perception of learning**			
1. I am encouraged to participate in class	1.77 ± 1.3	1.90 ± 1.2	1.77 ± 1.3
13. The teaching is student centered	1.44 ± 1.3	1.48 ± 1.3	1.33 ± 1.3
16. The teaching helps to develop my competence	1.43 ± 1.2	1.25 ± 1.3	1.17 ± 1.3
22. The teaching helps to develop my confidence	1.56 ± 1.3	1.53 ± 1.4	1.35 ± 1.3
24. The teaching time is utilized properly	1.83 ± 1.3	1.97 ± 1.4	1.75 ± 1.4
25. The teaching over emphasizes factual learning*	1.60 ± 1.2	1.56 ± 1.2	1.67 ± 1.3
38. I am clear about the learning objectives of the courses	1.99 ± 1.3	-	-
44. The teaching encourages me to be an active learner	1.62 ± 1.3	1.83 ± 1.4	1.48 ± 1.4
47. Long term learning is emphasized over short-term learning	1.73 ± 1.3	-	1.63 ± 1.4
48. The teaching is too teacher centered	1.79 ± 1.3	1.76 ± 1.2	1.70 ± 1.3
**Students’ perception of teachers**			
9. The teachers are authoritarian*	1.82 ± 1.3	1.54 ± 1.4	1.78 ± 1.4
32. The teachers provide constructive criticism	-	-	1.89 ± 1.4
**Students’ academic self-perception**			
5. Learning strategies which worked for me before continue to work even now	1.92 ± 1.4	1.72 ± 1.4	1.79 ± 1.4
21. I feel I am being well prepared for my profession	1.38 ± 1.2	1.40 ± 1.3	1.42 ± 1.3
26. Last year’s work has been a good preparation for this year’s work	1.88 ± 1.3	1.91 ± 1.2	1.89 ± 1.3
27. I am able to memorize all I need	1.80 ± 1.3	1.65 ± 1.3	1.64 ± 1.4
45. Much of what I have to learn seems relevant to a career in healthcare	-	1.98 ± 1.4	-
**Students’ perception of the atmosphere**			
11. The atmosphere is relaxed during the clinical teaching	1.71 ± 1.2	1.66 ± 1.1	1.78 ± 1.2
12. This school is well timetabled	1.47 ± 1.4	1.92 ± 1.4	1.72 ± 1.4
17. Cheating is a problem in the school*	-	-	1.61 ± 1.4
23. The atmosphere is relaxed during lectures	1.71 ± 1.3	1.82 ± 1.3	1.68 ± 1.3
30. There are opportunities for me to develop interpersonal skills	1.87 ± 1.3	1.86 ± 1.3	1.91 ± 1.4
34. The atmosphere is relaxed during seminars/tutorials	1.95 ± 1.2	-	1.86 ± 1.3
42. The enjoyment outweighs the stress of the courses	1.33 ± 1.3	1.43 ± 1.4	1.40 ± 1.4
43. The atmosphere motivates me as a learner	1.56 ± 1.3	1.82 ± 1.4	1.63 ± 1.5
49. I feel I am able to ask the questions I want	1.93 ± 1.3	1.80 ± 1.3	1.96 ± 1.4
**Students’ social self-perception**			
3. There is a good support system for students who get stressed	0.60 ± 0.9	0.63 ± 0.9	0.68 ± 1.0
4. I am too tired to enjoy the courses*	1.31 ± 1.4	1.33 ± 1.3	1.07 ± 1.2
14. I am rarely bored on the courses	1.12 ± 1.3	1.02 ± 1.2	1.33 ± 1.4
46. My accommodation in the school is pleasant	1.28 ± 1.1	1.66 ± 1.1	1.44 ± 1.1

^*^Negatively phrased items were coded in reverse so that the higher the score, the more positive the perception is

## Discussion

The current study aimed to provide the first evaluation of the learning environment at medical schools in Syria. Therefore, the DREEM inventory was used to measure the LE at the three largest medical schools in Syria, namely Damascus University, the University of Aleppo, and Tishreen University. The overall DREEM score at the three universities in total indicates that the learning environment had ‘plenty of problems.’ The mean scores for all students across all universities (*n* = 1774) as shown in Table 3 indicate that *Learning, Academic Self-perception, Atmosphere, Social Self-perception* had scores with a negative interpretation according to the grading rubric illustrated in Table 1. The perception of *Teachers'* score, on the other hand, indicated that medical schools are *moving in the right direction* in this domain. The most negatively perceived domains were students’ perception of *Learning* and *Social Self-perception* which DREEM scores were 45% and 43.5% of the total score respectively. Clinical students’ perception of the LE was significantly lower than their pre-clinical counterparts. Similarly, male students’ perception of the LE was significantly lower than female students.

A systematic review of DREEM studies published in 2018 showed that out of 98 studies, 79 (80.6%) scored within the range of 101–150 out of 200 (i.e. “*more positive than negative*”) [22]. Only seven DREEM studies on medical undergraduate education reported a DREEM score within the range of 51–100 (i.e. “*plenty of problems*”) [22]; most of these low-scoring medical schools were in Saudi Arabia, Yemen, Morocco, and Iran [10, 11, 23–25] and had a traditional curriculum. A comparison of DREEM scores between Syrian medical schools and other international medical schools is provided in Table 8, Supplementary file 1. Low DREEM scores were associated with traditional discipline-based curriculum, lecture-based teacher-centered approaches, and passive student learning. All of these elements are also present at Syrian medical schools [9]. Higher DREEM scores were observed in schools with a modern curriculum which included problem-based learning [26], system-based learning[27], and integrated curriculum [28]. Quality of life has also been found to be positively associated with the perception of the LE [29]. Although no studies evaluated the quality of life of medical students in Syria before, the war and economic crises imply poor quality of life and living conditions [30].

Several studies reported that students at the clinical stage viewed the LE more negatively than their pre-clinical counterparts [31–34] and this is consistent with our study findings. Clinical students perceived *Learning* and *Atmosphere* especially more negative than their pre-clinical counterparts. Clinical training in Syrian medical schools is unstructured and students are given little to no instructions on what to do in the hospitals. The unavailability of attending doctors and the huge number of medical students limit the engagement of each student with the doctor. Other factors that might make clinical students perceive the LE more negatively is the increased stress and the heavy pressure of studying for the Unified National Medical Examination which accounts for 50% of their cumulative average. In terms of differences between male and female students, the current research findings are in accord with recent studies indicating that LE is perceived more negatively by male students in comparison to female students [35, 36]. In the current study, male students scored significantly lower than females in their perception of the *Teachers* and *Atmosphere.* One possible explanation for this result is that faculty members-who are mostly males according to our knowledge-usually have a more empathetic attitude towards female medical students than their male counterparts, and this explanation has been proposed in a study on medical students in Egypt that found female students less likely than male students to report relationship problems with teachers as a source of stress [37]. It is beyond the scope of this study to investigate the differences between different healthcare fields; nevertheless, for comparison, the DREEM scores reported for medical schools in Syria were lower than their dental school counterparts [38]. DU and TU dental schools scored 105.9 and 111.1 in comparison to 100.8 and 97.8 found in the current study [38]; DU Pharmacy School scored even lower on the DREEM scale (mean score = 89.8) [39]

### Connecting the phenomenon to the context

The Syrian medical school context is still considered largely different than any other. First, Syria has been undergoing a civil war since 2011; the war caused major internal and external displacement, led to the deterioration of the quality of life and the population suffered from major physical, psychological, and social trauma. Second, the international sanctions have left Syria in a state of isolation, cutting all ties and relationships with other countries; in actuality, there was an undergoing curriculum reform at Damascus University Medical School that ceased due to the imposed sanctions in 2011 [8]. Third, the collapse of the Syrian economy and rampant inflation rates have resulted in poorer living conditions and difficulty in securing basic life needs [40, 41]. Major public medical schools such as DU, AU, and TU are under a lot of pressure, especially after the drastic increase in student intake due to the interruption of education at medical schools in conflict areas [9]. In addition, the university teaching capacity has been decreasing due to the exodus of a huge portion of the teaching staff [42]. Another factor that makes Syrian medical schools unique is that they are the only ones that maintained the use of native language-Arabic-as the main language of instruction in health profession education [43]. The taught content at medical schools in Syria is limited to the available Arabic study materials which are much poorer in quality and limited in quantity in comparison to their English counterparts. A previous study highlighted negative students’ attitudes towards Arabic as the language of instruction [44].

### What are the implications of having an unfavorable learning environment?

The current diagnosis of the learning environment at medical schools in Syria is worrying in many aspects. Educationally, students’ skills and knowledge attainment are bound to be affected [1]. Professionally, graduates might develop an unfavorable unsympathetic attitude towards patients [3]. Nationally, the negative LE might affect the retention rate of medical students which is already significantly low as many medical graduates leave their country after obtaining their degree. A study in 2018 reported that over 78% of medical students in Syria want to specialize outside Syria in countries such as Germany and the US [45]. In a country torn by war, there are many factors that might drive medical students to immigrate abroad including: job insecurity, financial burden, the economic crisis as well as fear for one’s personal safety [45][45]. Even though war and conflict-related factors were the major reason for medical students' exodus at the start of the Syrian conflict in 2011 [46], the deterioration of the learning environment might be a contributing factor to this exodus now in 2022. It had been previously discussed that one of the factors that drive Syrian medical students to specialize abroad was the quality of training [45]; teaching staff’s had a negative role which perpetuated students’ negative attitude towards staying in the country [45]. Medical students have a glimpse of what their working environment is like during their residency through the clinical rounds they have in 4^th^, 5^th^, and 6^th^ years of study; residents are poorly paid despite the long working hours they spend at the teaching hospitals [45, 47]. A study conducted in 2020 on the working and learning environment of resident dentists at Damascus University in Syria reported in detail the grim reality and unfortunate circumstances they deal with [17]. Resident dentists are usually left without the supervision of attending dentists who are few in number, frequently absent, and display little motivation towards teaching [17]. Residents strive to learn and largely depend on self-directed learning and the guidance of more senior peers [17]. Moreover, the management and maintenance of dental facilities were generally poor [17]. Taking into consideration the patient care pressure, long working hours, and the need for sophisticated and expensive medical equipment-which might not be always available, it is expected that the work environment at teaching hospitals is similarly negative if not worse than its dental counterpart. In certain places, residents have to purchase medical exam gloves, surgical masks, and other necessary medical tools that are not provided by the hospital to treat patients. All these unfortunate circumstances could contribute to the brain drain of the young healthcare workforce in Syria.

Despite the continuous deterioration of the medical schools’ capacity that manifested in the immigration of teaching staff and inability to provide maintenance to the current facilities nor fund expansion/renovation infrastructure projects, the medical schools are increasing their students’ yearly intake. The interruption of education in major public medical schools in conflict areas drastically increased the intake of students intake of medical schools in conflict-free zones [9], mainly Damascus University as can be seen in Table 2. The demand for health professionals is high in fragile contexts like Syria; nevertheless, the increase in medical students’ intake might always translate to an increase in health professionals or specialists especially when the majority of medical students have intentions to travel abroad for better living conditions. Moreover, increasing the students’ intake on the account of education quality might decrease the school output of competent doctors who are well-trained to respond effectively to the healthcare demands of the Syrian population [9]. Considering the possible negative implications of the insufficient teaching capacity to accommodate the large number of students on education quality and learning environment [15, 38], increasing the medical students’ yearly intake might be an indirect contributing factor to medical students' exodus from Syria.

### What’s next for the medical learning environment in Syria?

There are many obvious steps Syrian medical schools can take to improve the LE such as modernizing their teaching and reforming their curriculum. Nevertheless, the implementation of a true curriculum transformation cannot be done without a cohort of competent education experts and a facilitating institutional framework [8]. The second one is of utmost importance and is most difficult to achieve due to the fact that institutional policies are tied to University governance which is tied to national and legislative law that requires upper-level governmental approval prior to any change [8]. The only tried-and-true approach is the one that stems from committed and informed decision makers who are willing to make sacrifices and defy the law for achieving a true educational transformation [8].

### Strengths and limitations

The current study is considered the first of its kind in Syria. No evaluations of the learning environment in Syrian medical schools have been taken before. Gaining information on the medical education system at Syrian medical schools is difficult and a lot of bureaucratic obstacles are put to hinder any attempt to reveal or research any shortcomings or faults in the system. Syrian universities in general have a low tolerance for any kind of negative feedback, whether it was from students, clinical supervisors, or teachers. Another strength of this study is that it is to the extent of our knowledge considered the largest DREEM study in terms of sample size reported in the scientific literature [22]. The large sample size that covered the three major medical schools in Syria supports generalizability of our findings. On the other hand, the use of non-probability sampling methods is a limitation of the study. Due to problematic institutional policies and pragmatic reasons, collecting a random sample was not feasible in our context. The findings of this study could be used as a baseline reading for the learning environment at medical schools in Syria. Future curriculum reform plans in Syria can utilize this data in drawing a blueprint of the desirable educational climate. Educators and decision makers who are in similar fragile contexts could also make use of these findings in deducing the potential deficiencies in the learning environment at their own medical schools.

## Conclusion

This is the first DREEM study at medical schools in Syria. The findings of this evaluation provide a baseline reading and reveal the significant shortcomings in the medical learning environment in Syria, highlighting the importance of reconsidering a transformative curriculum reform that could address the outdated teaching and learning approaches. Educators should consider instituting policies to ensure that the educational climate is safe, supportive, and pleasant for students whose psycho-social wellbeing is compromised due to the long-lasting political and economic crises. Future research should focus on designing interventions that address the deficiencies in the learning environment as identified in this study. Some of these interventions could be establishing a support system or a mentoring scheme that connects students with mentors who have experience in providing psychosocial support as well as academic or professional advice. Reforming the traditional curriculum has been long advocated for and our study places further emphasis on it. Organizing conferences, seminars, and other events to raise awareness among faculty members, decision-makers and university leaders could incentivize a positive change in some of the defective policies hindering the development of a sound medical education environment in Syria.

## Supplementary Information

**Additional file 1: Table 6. **Cronbach's Alpha values for the 50-item DREEM inventory and each of its subscales** Table 7. **Mean score for each DREEM item categorized according to their domain with an ascending order** Table 8. **A comparison between medical schools in Syria and other international medical schools.

**Additional file 2.** Includes the Arabic version of the DREEM inventory which was used in this study; the English version is included as well.

## Data Availability

All data analysed and/or generated are available on figshare [21]. https://doi.org/10.6084/m9.figshare.21186034.v1
