# Supplementary material for: Revealing the significant shortcomings in the learning environment at the three largest medical schools in Syria: what’s next?
Source: BMC Med Educ. 2023 Jan 3;23:2. doi: 10.1186/s12909-022-03978-4 (PMC9809110; doi:10.1186/s12909-022-03978-4)
Supplement: Supplementary file 1 — Additional file 1: Table 6. Cronbach's Alpha values for the 50-item DREEM inventory and each of its subscales Table 7. Mean score for each DREEM item categorized according to their domain with an ascending order Table 8. A comparison between medical schools in Syria and other international medical schools. [file 12909_2022_3978_MOESM1_ESM.docx]

**Table 6.** Cronbach's Alpha values for the 50-item DREEM inventory and each of its subscales

|  | Valid cases | Excluded cases | No. of items | Cronbach’s Alpha |
| --- | --- | --- | --- | --- |
| Students’ perception of learning (SPL)  (max=48) | 1774 | 0 | 12 | 0.753 |
| Students’ perception of teachers (SPT)  (max=44) | 1773 | 1 | 11 | 0.828 |
| Students’ academic self-perceptions (ASP) (max=32) | 1772 | 2 | 8 | 0.725 |
| Students’ perception of atmosphere (SPA) (max=48) | 1772 | 2 | 12 | 0.804 |
| Students’ social self-perception (SSP) (max=28) | 1773 | 1 | 7 | 0.603 |
| DREEM total score (max=200) | 1769 | 5 | 50 | 0.928 |

**Table 7.** Mean score for each DREEM item categorized according to their domain with an ascending order.

| Items according to their subscales | Damascus University  (n=941) | University of Aleppo  (n=533) | Tishreen University (n=300) |
| --- | --- | --- | --- |
|  | Mean±SD | Mean±SD | Mean±SD |
| Students’ perception of learning (SPL) | | | |
| 1. I am encouraged to participate in class | 1.77±1.3 | 1.90±1.2 | 1.77±1.3 |
| 7. The teaching is often stimulating | 2.35±1.3 | 2.52±1.3 | 2.21±1.4 |
| 13. The teaching is student centered | 1.44±1.3 | 1.48±1.3 | 1.33±1.3 |
| 16. The teaching helps to develop my competence | 1.43±1.2 | 1.25±1.3 | 1.17±1.3 |
| 20. The teaching is well focused | 2.45±1.2 | 2.53±1.2 | 2.50±1.2 |
| 22. The teaching helps to develop my confidence | 1.56±1.3 | 1.53±1.4 | 1.35±1.3 |
| 24. The teaching time is utilized properly | 1.83±1.3 | 1.97±1.4 | 1.75±1.4 |
| 25. The teaching over emphasizes factual learning* | 1.60±1.2 | 1.56±1.2 | 1.67±1.3 |
| 38. I am clear about the learning objectives of the courses | 1.99±1.3 | 2.04±1.3 | 2.08±1.4 |
| 44. The teaching encourages me to be an active learner | 1.62±1.3 | 1.83±1.4 | 1.48±1.4 |
| 47. Long term learning is emphasized over short-term learning | 1.73±1.3 | 2.01±1.4 | 1.63±1.4 |
| 48. The teaching is too teacher centered* | 1.79±1.3 | 1.76±1.2 | 1.70±1.3 |
| Students’ perception of teachers (SPT) | | | |
| 2. The teachers are knowledgeable | 3.20±0.9 | 2.88±1.0 | 3.16±0.9 |
| 6. The teachers are patient with patients | 2.56±1.1 | 2.26±1.1 | 2.33±1.2 |
| 8. The teachers ridicule the students* | 2.47±1.3 | 2.34±1.3 | 2.24±1.4 |
| 9. The teachers are authoritarian* | 1.82±1.3 | 1.54±1.4 | 1.78±1.4 |
| 18. The teachers have good communication skills with patients | 2.62±1.1 | 2.41±1.0 | 2.39±1.2 |
| 29. The teachers are good at providing feedback to students | 2.44±1.2 | 2.32±1.2 | 2.21±1.3 |
| 32. The teachers provide constructive criticism | 2.09±1.3 | 2.23±1.3 | 1.89±1.4 |
| 37. The teachers give clear examples | 2.66±1.1 | 2.59±1.2 | 2.70±1.2 |
| 39. The teachers get angry during teaching sessions* | 2.17±1.3 | 1.95±1.2 | 2.03±1.3 |
| 40. The teachers are well prepared for their teaching sessions | 2.60±1.2 | 2.51±1.2 | 2.60±1.2 |
| 50. The students irritate the teachers* | 2.40±1.2 | 2.16±1.3 | 2.09±1.4 |
| Students’ academic self-perception (ASP) | |  |  |
| 5. Learning strategies which worked for me before continue to work even now | 1.92±1.4 | 1.72±1.4 | 1.79±1.4 |
| 10. I am confident about my passing this year | 2.45±1.3 | 2.75±1.2 | 2.45±1.4 |
| 21. I feel I am being well prepared for my profession | 1.38±1.2 | 1.40±1.3 | 1.42±1.3 |
| 26. Last year’s work has been a good preparation for this year’s work | 1.88±1.3 | 1.91±1.2 | 1.89±1.3 |
| 27. I am able to memorize all I need | 1.80±1.3 | 1.65±1.3 | 1.64±1.4 |
| 31. I have learned a lot about empathy in my profession | 2.95±1.1 | 3.09±1.0 | 2.95±1.2 |
| 41. My problem-solving skills are being well developed | 2.18±1.3 | 2.20±1.3 | 2.12±1.4 |
| 45. Much of what I have to learn seems relevant to a career in healthcare | 2.08±1.3 | 1.98±1.4 | 2.02±1.4 |
| Students’ perception of the atmosphere (SPA) | | | |
| 11. The atmosphere is relaxed during the clinical teaching | 1.71±1.2 | 1.66±1.1 | 1.78±1.2 |
| 12. This school is well timetabled | 1.47±1.4 | 1.92±1.4 | 1.72±1.4 |
| 17. Cheating is a problem in the school* | 2.40±1.3 | 2.80±1.2 | 1.61±1.4 |
| 23. The atmosphere is relaxed during lectures | 1.71±1.3 | 1.82±1.3 | 1.68±1.3 |
| 30. There are opportunities for me to develop interpersonal skills | 1.87±1.3 | 1.86±1.3 | 1.91±1.4 |
| 33. I feel comfortable in teaching sessions socially | 2.31±1.3 | 2.33±1.3 | 2.34±1.4 |
| 34. The atmosphere is relaxed during seminars/tutorials | 1.95±1.2 | 2.10±1.3 | 1.86±1.3 |
| 35. I find my experience disappointing* | 2.56±1.4 | 2.50±1.4 | 2.59±1.4 |
| 36. I am able to concentrate well | 2.70±1.1 | 2.55±1.1 | 2.69±1.2 |
| 42. The enjoyment outweighs the stress of the courses | 1.33±1.3 | 1.43±1.4 | 1.40±1.4 |
| 43. The atmosphere motivates me as a learner | 1.56±1.3 | 1.82±1.4 | 1.63±1.5 |
| 49. I feel I am able to ask the questions I want | 1.93±1.3 | 1.80±1.3 | 1.96±1.4 |
| Students’ social self-perception (SSP) | | | |
| 3. There is a good support system for students who get stressed | 0.60±0.9 | 0.63±0.9 | 0.68±1.0 |
| 4. I am too tired to enjoy the courses* | 1.31±1.4 | 1.33±1.3 | 1.07±1.2 |
| 14. I am rarely bored on the courses | 1.12±1.3 | 1.02±1.2 | 1.33±1.4 |
| 15. I have good friends in this school | 3.26±1.1 | 3.09±1.2 | 3.14±1.1 |
| 19. My social life is good | 2.39±1.3 | 2.52±1.3 | 2.53±1.4 |
| 28. I seldom feel lonely | 2.12±1.4 | 2.18±1.5 | 2.10±1.4 |
| 46. My accommodation in the school is pleasant | 1.28±1.1 | 1.66±1.1 | 1.44±1.1 |

* Negatively phrased items were coded in reverse so that the higher the score the more positive is the perception

**Table 8.** A comparison between medical schools in Syria and other international medical schools.

|  | Author and Year | Country | Sample | DREEM  (max. 200) |
| --- | --- | --- | --- | --- |
| Damascus University | Alfakhry et al (2022) | Syria | 941 | 100.8 |
| Tishreen University | Alfakhry et al (2022) | Syria | 300 | 97.8 |
| Aleppo University | Alfakhry et al (2022) | Syria | 533 | 101.3 |
| King’s College London[1] | Sideris et al (2016) | UK | 21 | 155.2 |
| Hellenic Medical Schools[1] | Sideris et al (2016) | Greece | 62 | 145.6 |
| The Deakin University School of Medicine[2] | Condon et al (2016) | Australia | 236 | 140.9 |
| Gulf Medical College[3] | Shehnaz et al (2012) | UAE | 44 | 135.4 |
| Sana’s University[4] | Al-Hazimi et al (2004) | Yemen | 179 | 100 |
| Hormozgan University of Medical Sciences[5] | Aghamolaei & Fazel (2010) | Iran | 182 | 99.6 |
| King Abdul Aziz University[6] | Imran et al (2016) | Saudi Arabia | 279 | 124.9 |

Underlined cells indicate a value of negative interpretation.

1. Sideris MC, Papalois AE, Athanasiou T, Dimitropoulos I, Theodoraki K, Dos Santos FS, Paparoidamis G, Staikoglou N, Pissas D, Whitfield PC: **Evaluating the educational environment of an international animal model-based wet lab course for undergraduate students**. *Annals of Medicine and Surgery* 2016, **12**:8-17.

2. Condon BP, Worley PS, Condon JR, Prideaux DJ: **Student academic performance in rural clinical schools: The impact of cohort size and competition**. *Medical teacher* 2017, **39**(3):262-268.

3. Shehnaz SI, Sreedharan J, Gomathi KG: **Faculty and students’ perceptions of student experiences in a medical school undergoing curricular transition in the United Arab Emirates**. *Sultan Qaboos University Medical Journal* 2012, **12**(1):77.

4. Al-Hazimi A, Zaini R, Al-Hyiani A, Hassan N, Gunaid A, Ponnamperuma G, Karunathilake I, Roff S, McAleer S, Davis M: **Educational environment in traditional and innovative medical schools: a study in four undergraduate medical schools**. *EDUCATION FOR HEALTH-ABINGDON-CARFAX PUBLISHING LIMITED-* 2004, **17**(2):192-203.

5. Aghamolaei T, Fazel I: **Medical students' perceptions of the educational environment at an Iranian Medical Sciences University**. *BMC medical education* 2010, **10**(1):1-7.

6. Imran M, Shamim MS, Baig M, Farouq M, Gazzaz ZJ, Al-Mutairi OM: **Tale of two cities: comparison of educational environment of two colleges (Jeddah and Rabigh) affiliated with one university**. *J Pak Med Assoc* 2016, **66**(3):316-319.
