# Supplementary material for: Revealing the significant shortcomings in the learning environment at the three largest medical schools in Syria: what’s next?
Source: BMC Med Educ. 2023 Jan 3;23:2. doi: 10.1186/s12909-022-03978-4 (PMC9809110; doi:10.1186/s12909-022-03978-4)
Supplement: Supplementary file 2 — Additional file 2. Includes the Arabic version of the DREEM inventory which was used in this study; the English version is included as well. [file 12909_2022_3978_MOESM2_ESM.docx]

**DREEM questionnaire for evaluating the learning environment**

The English and Arabic versions of the DREEM questionnaire were extracted from a previous Ph.D. thesis conducted at Dundee University [1].

Please indicate to what extent you agree/disagree with the statement below:

1. I am encouraged to participate during teaching sessions

- 0=strongly disagree
- 1=disagree
- 3=agree
- 4=strongly agree
- 2=unsure/doesn’t apply

2. The teachers are knowledgeable

- 0=strongly disagree
- 1=disagree
- 3=agree
- 4=strongly agree
- 2=unsure/doesn’t apply

3. There is a good support system for students who get stressed

- 0=strongly disagree
- 1=disagree
- 3=agree
- 4=strongly agree
- 2=unsure/doesn’t apply

4. I am too tired to enjoy the courses

- 0=strongly disagree
- 1=disagree
- 3=agree
- 4=strongly agree
- 2=unsure/doesn’t apply

5. Learning strategies which worked for me before continue to work even now

- 0=strongly disagree
- 1=disagree
- 3=agree
- 4=strongly agree
- 2=unsure/doesn’t apply

6. The teachers are patient with patients

- 0=strongly disagree
- 1=disagree
- 3=agree
- 4=strongly agree
- 2=unsure/doesn’t apply

7. The teaching is often stimulating

- 0=strongly disagree
- 1=disagree
- 3=agree
- 4=strongly agree
- 2=unsure/doesn’t apply

8. The teachers ridicule the students

- 0=strongly disagree
- 1=disagree
- 3=agree
- 4=strongly agree
- 2=unsure/doesn’t apply

9. The teachers are authoritarian

- 0=strongly disagree
- 1=disagree
- 3=agree
- 4=strongly agree
- 2=unsure/doesn’t apply

10. I am confident about my passing this year

- 0=strongly disagree
- 1=disagree
- 3=agree
- 4=strongly agree
- 2=unsure/doesn’t apply

11. The atmosphere is relaxed during the clinical teaching

- 0=strongly disagree
- 1=disagree
- 3=agree
- 4=strongly agree
- 2=unsure/doesn’t apply

12. This school is well timetabled

- 0=strongly disagree
- 1=disagree
- 3=agree
- 4=strongly agree
- 2=unsure/doesn’t apply

13. The teaching is student centered

- 0=strongly disagree
- 1=disagree
- 3=agree
- 4=strongly agree
- 2=unsure/doesn’t apply

14. I am rarely bored on the courses

- 0=strongly disagree
- 1=disagree
- 3=agree
- 4=strongly agree
- 2=unsure/doesn’t apply

15. I have good friends in this school

- 0=strongly disagree
- 1=disagree
- 3=agree
- 4=strongly agree
- 2=unsure/doesn’t apply

16. The teaching helps to develop my competence

- 0=strongly disagree
- 1=disagree
- 3=agree
- 4=strongly agree
- 2=unsure/doesn’t apply

17. Cheating is a problem in the school

- 0=strongly disagree
- 1=disagree
- 3=agree
- 4=strongly agree
- 2=unsure/doesn’t apply

18. The teachers have good communication skills with patients

- 0=strongly disagree
- 1=disagree
- 3=agree
- 4=strongly agree
- 2=unsure/doesn’t apply

19. My social life is good

- 0=strongly disagree
- 1=disagree
- 3=agree
- 4=strongly agree
- 2=unsure/doesn’t apply

20. The teaching is well focused

- 0=strongly disagree
- 1=disagree
- 3=agree
- 4=strongly agree
- 2=unsure/doesn’t apply

21. I feel I am being well prepared for my profession

- 0=strongly disagree
- 1=disagree
- 3=agree
- 4=strongly agree
- 2=unsure/doesn’t apply

22. The teaching helps to develop my confidence

- 0=strongly disagree
- 1=disagree
- 3=agree
- 4=strongly agree
- 2=unsure/doesn’t apply

23. The atmosphere is relaxed during lectures

- 0=strongly disagree
- 1=disagree
- 3=agree
- 4=strongly agree
- 2=unsure/doesn’t apply

24. The teaching time is utilized properly

- 0=strongly disagree
- 1=disagree
- 3=agree
- 4=strongly agree
- 2=unsure/doesn’t apply

25. The teaching over emphasizes factual learning

- 0=strongly disagree
- 1=disagree
- 3=agree
- 4=strongly agree
- 2=unsure/doesn’t apply

26. Last year’s work has been a good preparation for this year’s work

- 0=strongly disagree
- 1=disagree
- 3=agree
- 4=strongly agree
- 2=unsure/doesn’t apply

27. I am able to memorize all I need

- 0=strongly disagree
- 1=disagree
- 3=agree
- 4=strongly agree
- 2=unsure/doesn’t apply

28. I seldom feel lonely

- 0=strongly disagree
- 1=disagree
- 3=agree
- 4=strongly agree
- 2=unsure/doesn’t apply

29. The teachers are good at providing feedback to students

- 0=strongly disagree
- 1=disagree
- 3=agree
- 4=strongly agree
- 2=unsure/doesn’t apply

30. There are opportunities for me to develop interpersonal skills

- 0=strongly disagree
- 1=disagree
- 3=agree
- 4=strongly agree
- 2=unsure/doesn’t apply

31. I have learned a lot about empathy in my profession

- 0=strongly disagree
- 1=disagree
- 3=agree
- 4=strongly agree
- 2=unsure/doesn’t apply

32. The teachers provide constructive criticism

- 0=strongly disagree
- 1=disagree
- 3=agree
- 4=strongly agree
- 2=unsure/doesn’t apply

33. I feel comfortable in teaching sessions socially

- 0=strongly disagree
- 1=disagree
- 3=agree
- 4=strongly agree
- 2=unsure/doesn’t apply

34. The atmosphere is relaxed during seminars/tutorials

- 0=strongly disagree
- 1=disagree
- 3=agree
- 4=strongly agree
- 2=unsure/doesn’t apply

35. I find my experience disappointing

- 0=strongly disagree
- 1=disagree
- 3=agree
- 4=strongly agree
- 2=unsure/doesn’t apply

36. I am able to concentrate well

- 0=strongly disagree
- 1=disagree
- 3=agree
- 4=strongly agree
- 2=unsure/doesn’t apply

37. The teachers give clear examples

- 0=strongly disagree
- 1=disagree
- 3=agree
- 4=strongly agree
- 2=unsure/doesn’t apply

38. I am clear about the learning objectives of the courses

- 0=strongly disagree
- 1=disagree
- 3=agree
- 4=strongly agree
- 2=unsure/doesn’t apply

39. The teachers get angry during teaching sessions

- 0=strongly disagree
- 1=disagree
- 3=agree
- 4=strongly agree
- 2=unsure/doesn’t apply

40. The teachers are well prepared for their teaching sessions

- 0=strongly disagree
- 1=disagree
- 3=agree
- 4=strongly agree
- 2=unsure/doesn’t apply

41. My problem solving skills are being well developed

- 0=strongly disagree
- 1=disagree
- 3=agree
- 4=strongly agree
- 2=unsure/doesn’t apply

42. The enjoyment outweighs the stress of the courses

- 0=strongly disagree
- 1=disagree
- 3=agree
- 4=strongly agree
- 2=unsure/doesn’t apply

43. The atmosphere motivates me as a learner

- 0=strongly disagree
- 1=disagree
- 3=agree
- 4=strongly agree
- 2=unsure/doesn’t apply

44. The teaching encourages me to be an active learner

- 0=strongly disagree
- 1=disagree
- 3=agree
- 4=strongly agree
- 2=unsure/doesn’t apply

45. Much of what I have to learn seems relevant to a career in healthcare

- 0=strongly disagree
- 1=disagree
- 3=agree
- 4=strongly agree
- 2=unsure/doesn’t apply

46. My accommodation in the school is pleasant

- 0=strongly disagree
- 1=disagree
- 3=agree
- 4=strongly agree
- 2=unsure/doesn’t apply

47. Long term learning is emphasized over short-term learning

- 0=strongly disagree
- 1=disagree
- 3=agree
- 4=strongly agree
- 2=unsure/doesn’t apply

48. The teaching is too teacher centered

- 0=strongly disagree
- 1=disagree
- 3=agree
- 4=strongly agree
- 2=unsure/doesn’t apply

49. I feel I am able to ask the questions I want

- 0=strongly disagree
- 1=disagree
- 3=agree
- 4=strongly agree
- 2=unsure/doesn’t apply

50. The students irritate the teachers

- 0=strongly disagree
- 1=disagree
- 3=agree
- 4=strongly agree
- 2=unsure/doesn’t apply

**End of questions**

**استبيان DREEM لتقييم البيئة التعليمية**

اختر الاجابة التي تعكس وجهة نظرك من العبارات التالية:

1. أشعر بالتشجيع دائما للمشاركة في المناقشات داخل الفصل

o       0= غير موافق بشدة

o       1=غير موافق بتحفظ

o       3=موافق بتحفظ

o       4=موافق بشدة

o       2=غير متأكد/لا تنطبق عليك

2. يتصف المدرسون في هذه الكلية بسعة المعرفة

o       0= غير موافق بشدة

o       1=غير موافق بتحفظ

o       3=موافق بتحفظ

o       4=موافق بشدة

o       2=غير متأكد/لا تنطبق عليك

3. يوجد نظام دعم جيد للطلاب/الطالبات الذين يقعون تحت ضغط نفسي

o       0= غير موافق بشدة

o       1=غير موافق بتحفظ

o       3=موافق بتحفظ

o       4=موافق بشدة

o       2=غير متأكد/لا تنطبق عليك

4. أنا أشعر بالإرهاق لدرجة لا تمكنني من الاستمتاع بهذا الفصل الدراسي

o       0= غير موافق بشدة

o       1=غير موافق بتحفظ

o       3=موافق بتحفظ

o       4=موافق بشدة

o       2=غير متأكد/لا تنطبق عليك

5.أساليب الدراسة التي كنت أستخدمها سابقا لا زلت اجدها فعالة الان

o       0= غير موافق بشدة

o       1=غير موافق بتحفظ

o       3=موافق بتحفظ

o       4=موافق بشدة

o       2=غير متأكد/لا تنطبق عليك

6. يتحلى المدرسون بالصبر مع المرضى

o       0= غير موافق بشدة

o       1=غير موافق بتحفظ

o       3=موافق بتحفظ

o       4=موافق بشدة

o       2=غير متأكد/لا تنطبق عليك

7.غالبا ما يكون التعليم حافزا للمشاركة و الاهتمام

o       0= غير موافق بشدة

o       1=غير موافق بتحفظ

o       3=موافق بتحفظ

o       4=موافق بشدة

o       2=غير متأكد/لا تنطبق عليك

8.. يسخر المدرسون من الطلاب في هذه الكلية

o       0= غير موافق بشدة

o       1=غير موافق بتحفظ

o       3=موافق بتحفظ

o       4=موافق بشدة

o       2=غير متأكد/لا تنطبق عليك

9. المدرسون ذوو طابع يغلب عليه حب السيطرة

o       0= غير موافق بشدة

o       1=غير موافق بتحفظ

o       3=موافق بتحفظ

o       4=موافق بشدة

o       2=غير متأكد/لا تنطبق عليك

10. أشعر بالثقة بالنفس من اجتياز الامتحانات في هذه السنة

o       0= غير موافق بشدة

o       1=غير موافق بتحفظ

o       3=موافق بتحفظ

o       4=موافق بشدة

o       2=غير متأكد/لا تنطبق عليك

11. الوسط التعليمي أثناء الدراسة في أجنحة المرضى يبعث على الراحة

o       0= غير موافق بشدة

o       1=غير موافق بتحفظ

o       3=موافق بتحفظ

o       4=موافق بشدة

o       2=غير متأكد/لا تنطبق عليك

12. يتصف الجدول الدراسي في هذه الكلية بدقة المواعيد الزمنية

o       0= غير موافق بشدة

o       1=غير موافق بتحفظ

o       3=موافق بتحفظ

o       4=موافق بشدة

o       2=غير متأكد/لا تنطبق عليك

13. عملية التدريس هنا تركز على أن يكون الطالب المحور الرئيسي في عملية التدريس

o       0= غير موافق بشدة

o       1=غير موافق بتحفظ

o       3=موافق بتحفظ

o       4=موافق بشدة

o       2=غير متأكد/لا تنطبق عليك

14. نادرا ما أشعر بالملل في هذا الفصل الدراسي

o       0= غير موافق بشدة

o       1=غير موافق بتحفظ

o       3=موافق بتحفظ

o       4=موافق بشدة

o       2=غير متأكد/لا تنطبق عليك

15. لدي أصدقاء جيدين في هذه الكلية

o       0= غير موافق بشدة

o       1=غير موافق بتحفظ

o       3=موافق بتحفظ

o       4=موافق بشدة

o       2=غير متأكد/لا تنطبق عليك

16. ريقة التدريس في هذه الكلية تساعدني على تطوير كفاءتي

o       0= غير موافق بشدة

o       1=غير موافق بتحفظ

o       3=موافق بتحفظ

o       4=موافق بشدة

o       2=غير متأكد/لا تنطبق عليك

17. يعتبر الغش مشكلة متفشية في هذه الكلية

o       0= غير موافق بشدة

o       1=غير موافق بتحفظ

o       3=موافق بتحفظ

o       4=موافق بشدة

o       2=غير متأكد/لا تنطبق عليك

18. يتمتع المدرسون بمهارات جيده للاتصال و التخاطب مع المرضى

o       0= غير موافق بشدة

o       1=غير موافق بتحفظ

o       3=موافق بتحفظ

o       4=موافق بشدة

o       2=غير متأكد/لا تنطبق عليك

19. أعتبر حياتي الاجتماعية في هذه الكلية جيدة

o       0= غير موافق بشدة

o       1=غير موافق بتحفظ

o       3=موافق بتحفظ

o       4=موافق بشدة

o       2=غير متأكد/لا تنطبق عليك

20. يتصف التدريس بالتركيز المباشر على موضوع الدرس

o       0= غير موافق بشدة

o       1=غير موافق بتحفظ

o       3=موافق بتحفظ

o       4=موافق بشدة

o       2=غير متأكد/لا تنطبق عليك

21. أشعر بأن الدراسة في هذه الكلية أعدتني إعدادا جيدا لأداء متطلبات مهنتي

o       0= غير موافق بشدة

o       1=غير موافق بتحفظ

o       3=موافق بتحفظ

o       4=موافق بشدة

o       2=غير متأكد/لا تنطبق عليك

22. عملية التدريس في هذه الكلية تساعدني على تطوير ثقتي بنفسي كطالب

o       0= غير موافق بشدة

o       1=غير موافق بتحفظ

o       3=موافق بتحفظ

o       4=موافق بشدة

o       2=غير متأكد/لا تنطبق عليك

23. الجو العام أثناء المحاضرات يدعو للراحة و الطمأنينة

o       0= غير موافق بشدة

o       1=غير موافق بتحفظ

o       3=موافق بتحفظ

o       4=موافق بشدة

o       2=غير متأكد/لا تنطبق عليك

24. الوقت المخصص للتدريس يستغل استغلالا جيدا

o       0= غير موافق بشدة

o       1=غير موافق بتحفظ

o       3=موافق بتحفظ

o       4=موافق بشدة

o       2=غير متأكد/لا تنطبق عليك

25. تركز عملية التدريس بشكل كبير على تعلم الحقائق

o       0= غير موافق بشدة

o       1=غير موافق بتحفظ

o       3=موافق بتحفظ

o       4=موافق بشدة

o       2=غير متأكد/لا تنطبق عليك

26. يعتبر عملي خلال السنة الأكاديمية الماضية تحضيرا جيدا لهذه السنة

o       0= غير موافق بشدة

o       1=غير موافق بتحفظ

o       3=موافق بتحفظ

o       4=موافق بشدة

o       2=غير متأكد/لا تنطبق عليك

27. أشعر بمقدرتي على حفظ كل ما أريد

o       0= غير موافق بشدة

o       1=غير موافق بتحفظ

o       3=موافق بتحفظ

o       4=موافق بشدة

o       2=غير متأكد/لا تنطبق عليك

28. نادرا ما أحس بالوحدة في هذه الجامعة

o       0= غير موافق بشدة

o       1=غير موافق بتحفظ

o       3=موافق بتحفظ

o       4=موافق بشدة

o       2=غير متأكد/لا تنطبق عليك

29. يجيد المدرسون التعامل مع أسئلة الطلاب و استفساراتهم

o       0= غير موافق بشدة

o       1=غير موافق بتحفظ

o       3=موافق بتحفظ

o       4=موافق بشدة

o       2=غير متأكد/لا تنطبق عليك

30. تتوفر في هذه الكلية عدة فرص لتحسين أسلوبي في التعامل مع الاخرين

o       0= غير موافق بشدة

o       1=غير موافق بتحفظ

o       3=موافق بتحفظ

o       4=موافق بشدة

o       2=غير متأكد/لا تنطبق عليك

31. مهنتي علمتني كثيرا كيف أتعاطف مع الاخرين

o       0= غير موافق بشدة

o       1=غير موافق بتحفظ

o       3=موافق بتحفظ

o       4=موافق بشدة

o       2=غير متأكد/لا تنطبق عليك

32. النقد الذي يوجهه المدرسون للطلبه من النوع البناء

o       0= غير موافق بشدة

o       1=غير موافق بتحفظ

o       3=موافق بتحفظ

o       4=موافق بشدة

o       2=غير متأكد/لا تنطبق عليك

33. أشعر بالراحة من الناحية الاجتماعية في هذا الفصل

o       0= غير موافق بشدة

o       1=غير موافق بتحفظ

o       3=موافق بتحفظ

o       4=موافق بشدة

o       2=غير متأكد/لا تنطبق عليك

34. الجو التعليمي العام مريح خلال المحاضرات و المناقشات/ و حلقات البحث

o       0= غير موافق بشدة

o       1=غير موافق بتحفظ

o       3=موافق بتحفظ

o       4=موافق بشدة

o       2=غير متأكد/لا تنطبق عليك

35. أعتقد أن تجربتي الدراسية مخيبه للامال

o       0= غير موافق بشدة

o       1=غير موافق بتحفظ

o       3=موافق بتحفظ

o       4=موافق بشدة

o       2=غير متأكد/لا تنطبق عليك

36. لدي المقدرة على التركيز الجيد

o       0= غير موافق بشدة

o       1=غير موافق بتحفظ

o       3=موافق بتحفظ

o       4=موافق بشدة

o       2=غير متأكد/لا تنطبق عليك

37. يقوم المدرسون بتقديم اأمثلة واضحة خلال المحاضرات

o       0= غير موافق بشدة

o       1=غير موافق بتحفظ

o       3=موافق بتحفظ

o       4=موافق بشدة

o       2=غير متأكد/لا تنطبق عليك

38. أنا أفهم بوضوح الأهداف التعليمية لهذا الفصل الدراسي و ما هو المطلوب تعلمه بنهاية الفصل

o       0= غير موافق بشدة

o       1=غير موافق بتحفظ

o       3=موافق بتحفظ

o       4=موافق بشدة

o       2=غير متأكد/لا تنطبق عليك

39. يغضب المدرسون أثناء المحاضرات

o       0= غير موافق بشدة

o       1=غير موافق بتحفظ

o       3=موافق بتحفظ

o       4=موافق بشدة

o       2=غير متأكد/لا تنطبق عليك

40. يتصف المدرسون بالإعداد الجيد لمحاضراتهم

o       0= غير موافق بشدة

o       1=غير موافق بتحفظ

o       3=موافق بتحفظ

o       4=موافق بشدة

o       2=غير متأكد/لا تنطبق عليك

41. طورت دراستي في هذه الكلية مهاراتي في حل المشاكل/المسائل الدراسية

o       0= غير موافق بشدة

o       1=غير موافق بتحفظ

o       3=موافق بتحفظ

o       4=موافق بشدة

o       2=غير متأكد/لا تنطبق عليك

42. المتعة الدراسية تفوق التعب في هذا الفصل الدراسي

o       0= غير موافق بشدة

o       1=غير موافق بتحفظ

o       3=موافق بتحفظ

o       4=موافق بشدة

o       2=غير متأكد/لا تنطبق عليك

43. الجو العام للدراسة في هذه الكلية يشجعني كطالب

o       0= غير موافق بشدة

o       1=غير موافق بتحفظ

o       3=موافق بتحفظ

o       4=موافق بشدة

o       2=غير متأكد/لا تنطبق عليك

44. في هذه الكلية يشجع التدريس على أن أكون طالب علم نشط و فعال

o       0= غير موافق بشدة

o       1=غير موافق بتحفظ

o       3=موافق بتحفظ

o       4=موافق بشدة

o       2=غير متأكد/لا تنطبق عليك

45. يبدو لي أن الكثير مما أتعلمه يتعلق بمهامي المستقبلة في مجال العناية الصحية

o       0= غير موافق بشدة

o       1=غير موافق بتحفظ

o       3=موافق بتحفظ

o       4=موافق بشدة

o       2=غير متأكد/لا تنطبق عليك

46. سكن جامعي مريح بشكل عام

o       0= غير موافق بشدة

o       1=غير موافق بتحفظ

o       3=موافق بتحفظ

o       4=موافق بشدة

o       2=غير متأكد/لا تنطبق عليك

47. تركز عملية التعليم على التعليم على المدى الطويل بدلا من التعليم على المدى القصير

o       0= غير موافق بشدة

o       1=غير موافق بتحفظ

o       3=موافق بتحفظ

o       4=موافق بشدة

o       2=غير متأكد/لا تنطبق عليك

48. تركز عملية التدريس على أن يكون المدرس هو المحور الرئيس في عملية التدريس

o       0= غير موافق بشدة

o       1=غير موافق بتحفظ

o       3=موافق بتحفظ

o       4=موافق بشدة

o       2=غير متأكد/لا تنطبق عليك

49. أشعر انه باستطاعتي توجيه أي سؤال أريد في هذه الكلية

o       0= غير موافق بشدة

o       1=غير موافق بتحفظ

o       3=موافق بتحفظ

o       4=موافق بشدة

o       2=غير متأكد/لا تنطبق عليك

50. كثيرا ما يستثير الطلاب غضب المدرسين في هذه الكلية

o       0= غير موافق بشدة

o       1=غير موافق بتحفظ

o       3=موافق بتحفظ

o       4=موافق بشدة

o       2=غير متأكد/لا تنطبق عليك

**نهاية الاستبيان**

1. Al-Qahtani MFM: **Approaches to study and learning environment in medical schools with special reference to the Gulf countries**. University of Dundee Dundee, UK; 1999.
